# Novel Unexpected Reconstructions of (100) and (111) Surfaces of NaCl: Theoretical Prediction

**DOI:** 10.1038/s41598-019-50548-8

**Published:** 2019-10-03

**Authors:** Alexander G. Kvashnin, Dmitry G. Kvashnin, Artem R. Oganov

**Affiliations:** 1 Skolkovo Institute of Science and Technology, Skolkovo Innovation Center, 3 Nobel Street, Moscow, 121205 Russia; 2 Moscow Institute of Physics and Technology, 9 Institutsky Pereulok, Dolgoprudny, 141700 Russia; 3 Emanuel Institute of Biochemical Physics RAS, 4 Kosigina Street, Moscow, 119334 Russia; 4 National University of Science and Technology MISIS, 4 Leninskiy Prospekt, Moscow, 119049 Russia; 5 International Center for Materials Discovery, Northwestern Polytechnical University, Xi’an, 710072 China

**Keywords:** Atomistic models, Two-dimensional materials, Structural properties

## Abstract

We have predicted stable reconstructions of the (100) and (111) surfaces of NaCl using the global optimization algorithm USPEX. Several new reconstructions, together with the previously reported ones, are found. For the cleaved bare (100) surface, pure Na and pure Cl are the only stable surface phases. Our study of the (111) surface shows that a newly predicted Na_3_Cl-(1 × 1) reconstruction is thermodynamically stable in a wide range of chlorine chemical potentials. It has a sawtooth-like profile where each facet reproduces the (100) surface of rock-salt NaCl, hinting on the preferred growth of the (100) surface. We used Bader charge analysis to explain the preferable formation of this sawtooth-like Na_3_Cl-(1 × 1) reconstruction of the (111) surface of NaCl. We find that at a very high chemical potential of Na, the polar (and normally absent) (111) surface becomes part of the equilibrium crystal morphology. At both very high and very low chemical potentials of Cl, we predict a large decrease of surface energy and fracture toughness (the Rehbinder effect).

## Introduction

One of the simplest and most thoroughly studied ionic crystals is sodium chloride, also known as table salt. It is well established that NaCl has a B1 (“rock-salt”) structure that transforms into the CsCl-type structure (the B2 phase) at about 30 GPa and room temperature^1^. A number of theoretical^2^ and experimental^3^ studies confirm that only these two phases exist in the 0–300 GPa pressure range. At a negative pressure, a wurtzite-type phase of NaCl was predicted^4^. Several stable Na_x_Cl_y_ phases of various unusual stoichiometries were discovered at pressures above 22 GPa in ref.^5^. Two of these exotic compounds, Na_3_Cl and Na_2_Cl, were found at normal conditions as stable 2D phases on a graphene substrate^6^.

In contrast to the bulk case, a completely different situation may occur at low dimensions, e.g. in thin films and on surfaces. The available reference data show that the most energetically favorable surface of NaCl is (100)^7–9^. This was confirmed by a number of experimental and theoretical studies, where thin films of sodium chloride with the (100) surface orientation were grown on various substrates, namely Cu(001)^10,11^, Cu(110)^12^, Cu(111)^13–19^, Cu(311)^20^, Cu(221)^20,21^, Ni(001)^11^, Ag(001)^19,22–24^, Ag(111)^19,25,26^, Au(111) and Au(111)-($22\times \sqrt{3}\,$)^27^. In addition, it was shown by Kiguchi *et al*.^11^ that the interaction between an alkali metal halide film and substrate is weak and the substrate does not influence the atomic structure of the grown films. Perhaps increasing the interaction between the substrate and film by selecting a particular substrate may lead to the formation of NaCl films with unusual structures.

Recent experiments on ultrathin films of various compounds show that thin films of zinc oxide (ZnO) terminated by a polar (0001) surface undergo a spontaneous splitting into graphene-like layers^28,29^. Moreover, graphene-like thin films of silicon carbide (SiC) and aluminum nitride (AlN) were also synthesized^30,31^. Detailed theoretical studies of the graphitization of thin films with different types of crystal structure, including NaCl with (111) surface, were reported^32–34^. There are also several experimental studies of the growth of NaCl films corresponding to the (111) surface on various substrates. In ref.^35^, the growth of NaCl(111) on the GaAs(111) surface was carried out using molecular beam epitaxy. As a result, an anomalous and unstable NaCl film with the (111) surface was obtained. Another appropriate substrate for growing alkali halides is silicon^36,37^. Reconstructed surfaces of Si(100)-(2 × 1)^36^ and Si(111)-(7 × 7)^37^ were used as substrates for growing bilayer NaCl films with the (100) surface^36^ and thin islands of KCl with both the (111) and (100) surfaces^37^. All the experimental evidence proves the energetic preference of the (100) surface of NaCl compared to the (111) surface, which, however, can also be obtained with sufficient care.

In all the above mentioned theoretical works the 2D films and surfaces were modeled assuming the fixed structure and composition equivalent to the bulk, without accounting for reconstructions and the effect of chemical potentials.

Here we search for thermodynamically stable reconstructions of the (100) and (111) surfaces of NaCl. We use the evolutionary algorithm USPEX^38–41^, which makes it possible to determine stable reconstructions and predict the conditions of their formation. Several interesting reconstructions of the NaCl(111) surface were found and their stability conditions determined.

## Results and Discussion

**Figure 1:**
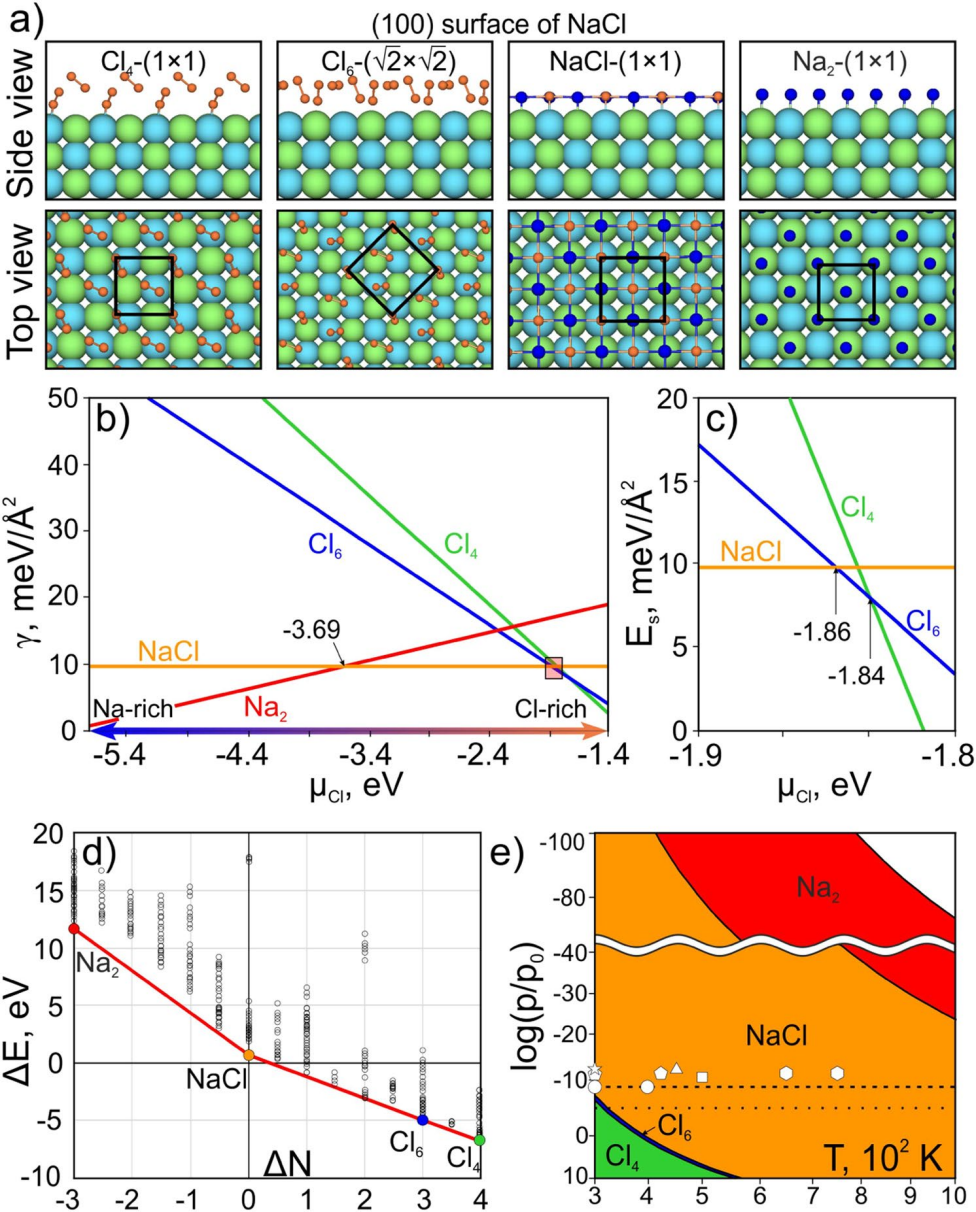
(**a**) The predicted reconstructions of the NaCl(100) surface. The chlorine atoms are shown in green, sodium atoms — in light blue. The surface atoms of chlorine and sodium are shown in orange and blue, respectively. (**b**) Surface energy as a function of µ_Cl_. (**c**) The enlarged region for the µ_Cl_ range from −1.8 eV to −1.9 eV. (**d**) Convex hull diagram, where solid circles indicate thermodynamically stable reconstructions; the colors of the circles correspond to the colors of the lines in (**b**). (**e**) Phase diagram of the NaCl(100) surface. The experimental conditions of the heteroepitaxial growth of the (100) surface on various substrates are taken from refs^10,11,19,20,22,52^ and represented by various white shapes.

First we study the (100) surface of NaCl, for which we predicted several thermodynamically stable reconstructions shown in Fig. 1a. We name the predicted reconstructions using the stoichiometry difference between the whole structure and substrate. This nomenclature is standard and was used in a number of previous works^42,43^. Four stable reconstructions were predicted, Cl_6_-($\sqrt{2}$ × $\sqrt{2}$), Cl_4_-(1 × 1), NaCl-(1 × 1), and Na_2_-(1 × 1), of which only one has both sodium and chlorine in its surface composition, while the rest of them have either pure chlorine or pure sodium surfaces (Fig. 1a).

The stability of the predicted reconstructions and its dependence on the chemical potential of chlorine was investigated in more details by calculating the surface energy diagram (Fig. 1b). The widest stability region with µ_Cl_ between −1.86 eV and −3.69 eV belongs to the unreconstructed NaCl(100) surface, which is perfectly consistent with the experimental evidence^13,14,44,45^. There are two reconstructions that can be formed under conditions of a chlorine excess, Cl_6_-($\sqrt{2}$ × $\sqrt{2}$) and Cl_4_-(1 × 1) (Fig. 1a). It is important that these chlorine-rich reconstructions are stable in very narrow ranges of chlorine chemical potentials (Fig. 1c) corresponding to the chlorine-rich environmental conditions. Another reconstruction, Na_2_-(1 × 1), can be formed in a lack of chlorine (Fig. 1a), and can be described as a layer of NaCl adsorbed on the NaCl(100) surface. At very low chemical potentials of chlorine (i.e., very high chemical potentials of Na) and at very high chemical potentials of chlorine, the energy of the (100) surface is greatly diminished (Fig. 1b). We recall that surface energy is related to fracture toughness K_g_ ^46^:

1 $${K}_{g}=2\sqrt{\gamma G/(1-\upsilon )},$$

where γ is the surface energy, G is the shear modulus, υ is the Poisson’s ratio of the material.

Therefore, we expect that NaCl will become much more brittle when immersed in gaseous or liquid Na or Cl, the phenomenon known as the Rehbinder effect^47–50^. Normally, the Rehbinder effect occurs because of a decrease in surface energy due to wetting; in our case it takes place because of the formation of new surface reconstructions that significantly reduce surface energy, which leads to a reduction in fracture toughness.

The calculated convex hull diagram is shown in Fig. 1d. A strong cusp corresponding to the nonreconstructed NaCl(100) surface indicates its stability in a wide range of chlorine chemical potentials, as seen in the surface diagram (Fig. 1b).

The calculated pressure-temperature phase diagram of the NaCl(100) surface shown in Fig. 1e makes it possible to predict the environmental conditions (partial chlorine pressure and temperature) suitable for the formation of a particular reconstruction. Both partial chlorine pressure and temperature are parts of the expression for chemical potential:

2 $${\mu }_{Cl}=\frac{1}{2}[{E}_{C{l}_{2}}+{G}_{C{l}_{2}}(T,\,{P}_{0})+{k}_{B}Tln(\frac{P}{{P}_{0}})]=\frac{1}{2}{E}_{C{l}_{2}}+\Delta {\mu }_{Cl}(T,\,P),$$

where ${G}_{C{l}_{2}}(T,\,{P}_{0})$ is the calculated Gibbs free energy of the gas of chlorine molecules at a certain temperature and pressure, which agrees well with the data from the thermodynamic database^51^. The details of the calculations and comparison with the reference data are shown in Supporting Information Table S1.

The calculated phase diagram (Fig. 1e) shows stability of the Cl_4_-(1 × 1) reconstruction at a chlorine partial pressure >10^−5^ bar (dotted horizontal line in Fig. 1e) and temperatures <320 K. The phase boundary between the Cl_4_-(1 × 1) and Cl_6_-(2 × 1) reconstructions in Fig. 1e corresponds to µ_Cl_ = −1.84 eV. Each phase boundary was plotted using Eq. (2) with the corresponding value of the chemical potential of chlorine. Increasing the temperature to 320 K at the chlorine partial pressure of 10^−5^ bar (dotted horizontal line) will lead to the formation of the Cl_6_-(2 × 1) reconstruction with a very narrow stability region, shown in blue in Fig. 1e. Further increase in temperature to 400 K will lead to the formation of a cleaved bare NaCl(100) surface that has the largest stability field (orange region in Fig. 1e). The chlorine partial pressure of 4 × 10^−9^ bar (dashed horizontal line in Fig. 1e) corresponds to the experimental pressure conditions of the homoepitaxial NaCl growth, where the formation of the cleaved bare (100) surface was observed^52^. The temperatures of 300 K and 400 K from the experiment are shown by two white circles^52^. Our simulation is consistent with these experiments, where at chlorine pressures <10^−10^ bar the cleaved bare NaCl(100) surface is stable in the whole temperature range up to the melting point of NaCl (~1100 K)^52^. Very low chlorine pressures <10^−20^ bar lead to conditions of sodium excess with µ_Cl_ > −3.69 eV. Such a low pressure, as well as high temperatures >900 K, lead to the formation of the Na_2_-(1 × 1) reconstruction, shown in red in Fig. 1e.

The NaCl(111) surface is much more interesting. It is polar and has a higher surface energy γ compared to the (100) surface: the values of 0.024 eV/Å^2^ (ref.^53^) or 0.033 eV/Å^2^ (ref.^54^) were computed for the (111) surface *vs*. 0.01 eV/Å^2^ computed for the (100) surface^53^. Our calculations give similar values for the surface energies of nonreconstructed (100) and (111) surfaces: 0.010 eV/Å^2^ and 0.030 eV/Å^2^, respectively, and these values are independent of the chemical potentials (the energies of reconstructed surfaces will, in general, depend on the chemical potentials, according to Eq. (6)). The polarity of the (111) surface leads to the formation of surprising surface phases like freestanding ultrathin NaCl films^32,34^. Motivated by this, we tried to answer the following questions: How does the (111) surface change at nanoscale? Can we find any stable unexpected reconstructions?

**Figure 2:**
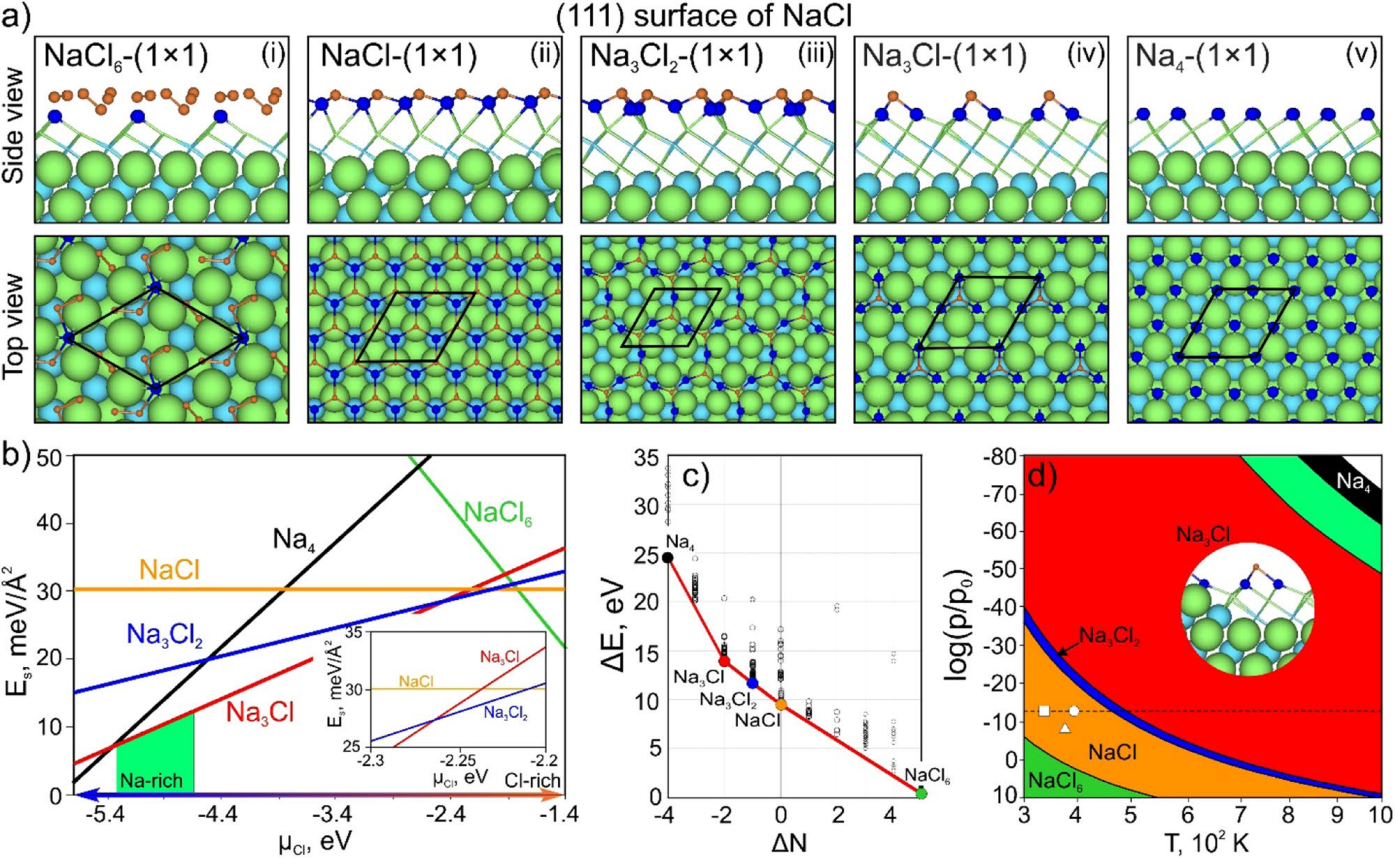
(**a**) Atomic structures of the predicted stable reconstructions of the (111) surface of NaCl: NaCl_6_-(1 × 1), NaCl-(1 × 1), Na_3_Cl_2_-(1 × 1), Na_3_Cl-(1 × 1), and Na_4_-(1 × 1). (**b**) Surface energy as a function of µ_Cl_. The region of µ_Cl_ = −2.2 eV to −2.3 eV is shown enlarged in an inset. (**c**) Convex hull diagram, where solid circles indicate the thermodynamically stable surface phases; the colors of the circles correspond to the colors of the lines in (**b**). (**d**) Phase diagram of the (111) surface of NaCl. The dashed horizontal line corresponds to ultrahigh vacuum conditions (10^−13^ bar). Reference data on the experimental conditions of NaCl(111) growth on Si(100)-(2 × 1)^36^, Si(111)-(7 × 7)^37^, and GaAs(111)^35^ substrates are represented by the small circle, square, and triangle, respectively. The light green region in (**b**) indicates the conditions at which Na_3_Cl-(1 × 1) appears on the crystal shape according to the Wulff construction (in this case, it corresponds to γ(111)/γ(100) < $\sqrt{3}$).

To answer these questions, we performed a search and found five stable reconstructions of the NaCl(111) surface, namely, NaCl_6_-(1 × 1), NaCl-(1 × 1), Na_3_Cl_2_-(1 × 1), Na_3_Cl-(1 × 1), and Na_4_-(1 × 1), shown in Fig. 2a. As all these reconstructions have a 1 × 1 unit cell, we omit the “-(1 × 1)” part in their names below. There is one reconstruction with a high concentration of chlorine (NaCl_6_ in panel (i) of Fig. 2a). Despite the additional Cl_2_ molecules that chlorine forms on this surface, the NaCl_6_ reconstruction has a corrugated “sawtooth-like” profile with sodium atoms (shown in dark blue) located at each vertex. Each facet of this surface reproduces the (100) surface of NaCl.

The nonreconstructed (111) NaCl surface is found to be slightly graphitized (panel (ii) in Fig. 2a). The top layer moves away from the substrate, forming a corrugated graphene-like structure. The graphitization process like this helps to remove the surface dipole as was predicted earlier for freestanding ultrathin NaCl films with (111) surfaces^32^. However, here the substrate plays an important role leading to the corrugation of the outer layer in contrast to the freestanding films, though the distance between the layer and substrate is the same as for the thin graphitic NaCl films^32^ and equals 3.7 Å (panel (ii) of Fig. 2a). A very loose structure (top view in panel (iii) of Fig. 2a) of the Na_3_Cl_2_ reconstruction is stable in a very narrow range of chlorine chemical potentials, from −2.26 eV to −2.23 eV (inset in Fig. 2b). This reconstruction can be considered as transient between NaCl and Na_3_Cl, where the regular structure of NaCl is lost.

In the Na_3_Cl surface reconstruction (panel (iv) of Fig. 2a), the chlorine atoms in the outer layer form a sawtooth-like surface where each facet reproduces the (100) surface of NaCl. This surface is similar to NaCl_6_ (if we disregard Cl_2_) as both display the facets of (100). During the growth of the (111) surface of NaCl, a more stable surface (in this case (100)) will grow slower than (111) and a sawtooth-like surface with (100) facets will form. The Na_4_ reconstruction represents the sodium-terminated (111) surface of NaCl (panel (v) in Fig. 2a).

Thermodynamic stability of the predicted reconstructions is shown in the computed surface energy diagram (Fig. 2b). The stability field of the chlorine-rich reconstruction NaCl_6_ is relatively narrow, in the range of µ_Cl_ from −1.41 eV to −1.83 eV (green line in Fig. 2b). A chlorine-rich atmosphere is needed to obtain this reconstruction. Interestingly, the graphitic-like reconstruction of the (111) surface of NaCl is stable in the range of chlorine chemical potentials from −1.83 eV to −2.21 eV (orange line in Fig. 2b). The cleaved (111) surface of NaCl reconstructs to a graphene-like surface to compensate the surface dipole component normal to the (111) surface caused by the polarity of the surface (due to the alignment of atoms within one atomic plane). This is similar to the graphitization of thin freestanding NaCl films^32^. We found that the cleaved (111) nongraphitized surface is located very close to the convex hull line, being just 0.04 eV above it.

The Na_3_Cl_2_ reconstruction has a very narrow stability field at µ_Cl_ from −2.22 eV to −2.27 eV, which can also be seen from the convex hull diagram in Fig. 2c. The slope of the hull almost does not change, with the Na_3_Cl_2_ reconstruction point located almost exactly on the straight line between the Na_3_Cl and NaCl reconstructions (Fig. 2c).

The widest stability field belongs to the Na_3_Cl reconstruction. The convex hull has a strong cusp corresponding to this surface phase (Fig. 2c), which is stable in a wide range of chlorine chemical potentials, from −2.27 eV to −5.36 eV.

Next we define the range of values of the chlorine chemical potential at which the (111) surface will appear on the equilibrium shape of the crystal using the Wulff construction^55^. In a single crystal of NaCl with predominant (100) surfaces, the (111) surface will exist only if ${\gamma }^{(111)}({\mu }_{Cl})/{\gamma }^{(100)}({\mu }_{Cl}) < \sqrt{3}$. Using the calculated surface energies as a function of chlorine chemical potential, for reconstructions of the (100) and (111) surfaces we found that at µ_Cl_ <−4.62 eV the surface energy of Na_3_Cl-(1 × 1) satisfies the above condition. However, at µ_Cl_ < −5.36 eV the stable phase of the (100) surface changes: the Na_2_-(1 × 1) reconstruction becomes stable (Fig. 1b). Taking this into account, we conclude that it is possible to obtain the Na_3_Cl-(1 × 1) reconstruction of the (111) surface on the equilibrium shape of a NaCl crystal at −5.36 eV < µ_Cl_ < −4.62 eV (Fig. 2b). The Wulff constructions for several values of µ_Cl_ are shown in Fig. S1 (see Supporting Information).

Let us consider the ${P}_{C{l}_{2}}-T$ phase diagram for all the studied reconstructions (Fig. 2d). Here, the dashed line denotes the pressure of 10^−13^ bar, which corresponds to ultrahigh vacuum conditions. The conditions of the NaCl(111) surface formation on Si(100)-(2 × 1)^36^ and Si(111)-(7 × 7)^37^ substrates are within the calculated stability region of the heteroepitaxial growth of the (111) surface (marked by a small circle and square in Fig. 2d). The triangle in Fig. 2d indicates the chlorine pressure of 5 × 10^−8^ bar and the temperature of ~110 °C, corresponding to the experimental conditions from ref.^35^. According to the calculated phase diagram, the formation of the Na_3_Cl reconstruction should occur at high-temperature conditions (red region in Fig. 2d). We have translated the range of chlorine chemical potentials at which the (111) surface will appear into the temperature and partial pressure of chlorine (light green regions in Fig. 2d). The results show that the Na_3_Cl(111) reconstruction can be obtained as an equilibrium surface only at high temperatures >700 K and extremely low chlorine pressures (Fig. 2d).

**Figure 3:**
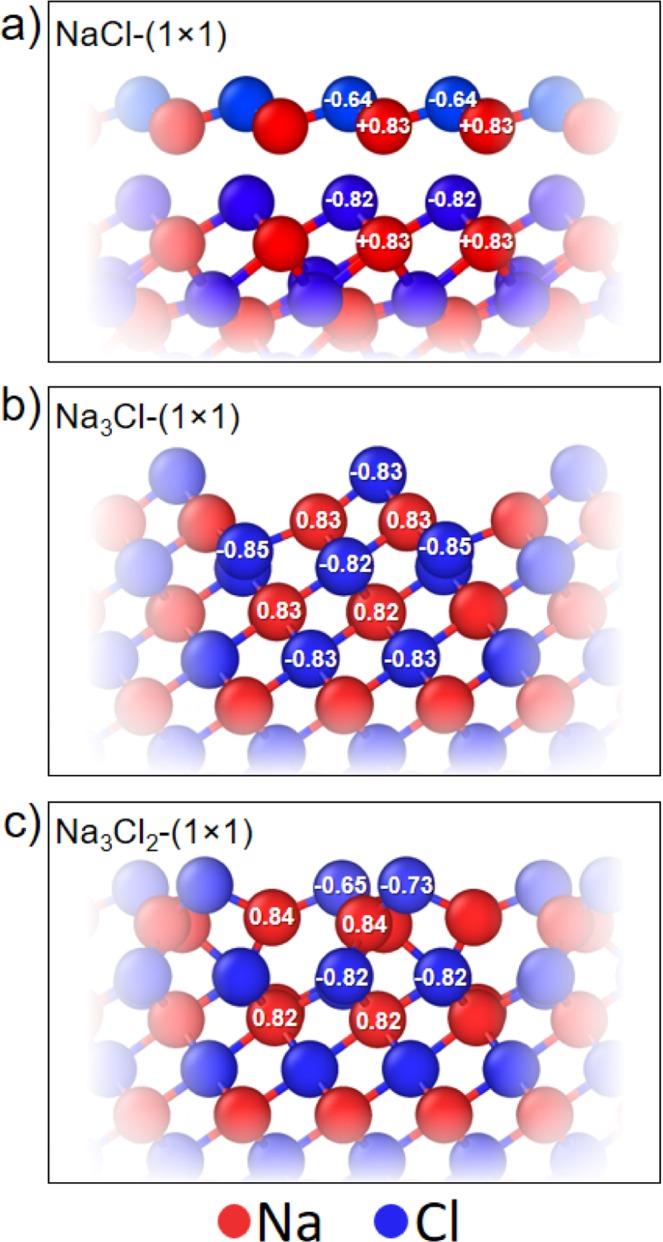
Bader charges (to be compared with the bulk values of +0.83 for Na and −0.83 for Cl) for the (111) surface reconstructions of NaCl including (**a**) NaCl-(1 × 1), (**b**) Na_3_Cl-(1 × 1), and (**c**) Na_3_Cl_2_-(1 × 1). The red and blue colors depicted sodium and chlorine atoms.

It is important that all the reconstructions of the (111) surface are polar (i.e. a different number of cations and anions is observed on the surface). According to the previous study of thin NaCl films^32^, to reduce the surface energy, the structure of thin films should minimize the dipole moment component normal to the surface. We studied the (111) surface reconstructions using Bader analysis (Figure 3), which gave the atomic charges in the bulk NaCl equal to +0.83*e* and −0.83*e* for the sodium and chlorine atoms, respectively. Fig. 3 shows the deviations of charges of the surface atoms from these values.

We found that the Na_3_Cl-(1 × 1) reconstruction has no charge difference relative to the bulk values (Fig. 3b), while for the surface atoms of the NaCl-(1 × 1) and Na_3_Cl_2_-(1 × 1) reconstructions the charges are clearly different. The obtained charges of the surface Cl atoms were −0.66*e* and −0.69*e* on average (Fig. 3a,c) for the NaCl-(1 × 1) and Na_3_Cl_2_-(1 × 1) surface reconstructions, respectively, which is 0.17*e* and 0.13*e* lower in absolute values than that for the Cl atoms in the bulk NaCl, while Na atoms have the same charges as in the bulk. This reduces surface dipole. We should also mention, that these reconstructions are metallic.

## Conclusions

Using the global optimization algorithm USPEX, we have studied a textbook case of the (100) and (111) surfaces of NaCl. The former is viewed as an archetypal “simple” surface: stable, stoichiometric, nonreconstructing, and nonpolar. The latter is regarded as a classical polar surface, normally unstable. Our results paint a much more complex picture. At extreme values of chemical potentials, we have found the deposition of Na (at low chemical potentials of Cl) and Cl (at high chemical potentials of Cl) and very low surface energies, which will lead to the embrittlement of NaCl in Na-rich and Cl-rich environments, a manifestation of the Rehbinder effect. On the (111) surface, we predict exotic stable surface compounds, NaCl_6_-(111) and Na_3_Cl-(111), which stabilize this polar surface, reducing its dipole moment. The new Na_3_Cl-(1 × 1) reconstruction is found to have the widest range of stability among all the other compounds considered here for the (111) surface. Moreover, contrary to conventional thinking, we find from the Wulff theorem that the (111) surface with the Na_3_Cl-(1 × 1) compound can be stable on the equilibrium shape in Na-rich conditions.

### Computational details

Stable reconstructions of the NaCl surfaces were predicted using the first-principles evolutionary algorithm (EA) as implemented in the USPEX code^38–40^, in its adaptation for surfaces^41^. In the presented calculations we included all the surface supercells up to index 4. Here, evolutionary searches were combined with structure relaxations using density functional theory (DFT)^56,57^ within the generalized gradient approximation (Perdew-Burke-Ernzerhof functional)^58^ and projector-augmented wave method^59,60^ as implemented in the VASP^61–63^ package. The plane-wave energy cutoff of 500 eV and *k*-mesh of $2\pi \times 0.05{\AA }^{-1}\,$resolution ensure excellent convergence of the energy difference, stresses, and forces. Monopole, dipole, and quadrupole corrections were taken into account using the method discussed in refs^64,65^. During the structure search, the first generation was produced randomly, while subsequent generations were obtained by applying 40% heredity, 10% softmutation, and 20% transmutation operations, respectively, with 30% of each new generation produced using the random symmetric algorithm^66^. Each of the studied supercells under study contained a vacuum layer with the thickness of 20 Å and a 6-Å-thick substrate slab of three NaCl layers for the (001) surface, with the topmost 3-Å layer allowed to relax, while for the (111) surface a substrate slab of ~8 Å was chosen, with the topmost 3-Å layer allowed to relax.

To perform variable-composition searches for stable surface reconstructions, it is important to set the boundary values of the physically allowed chemical potentials, which are related to the free energies of bulk Na, Cl_2_ gas, and bulk NaCl. The surface energies of the predicted reconstructions were calculated as

3 $$\gamma =\frac{1}{N}[{E}_{tot}-{E}_{ref}-\sum _{i}{n}_{i}{\mu }_{i}],$$

where *E*_*tot*_ is the total energy of the whole system, *E*_*ref*_ is the reference energy of the substrate (the unreconstructed cleaved (100) or (111) surface), *n*_*i*_ is the number of additional atoms of type *i* (Na or Cl) on the substrate, *μ*_*i*_ is the chemical potential of the atom of type *i*, *N* = *m* × *n* for an *m* × *n* surface supercell (serves as a normalization factor).

In the case of NaCl, Eq. (3) becomes

4 $$\gamma (T,\,P)=\frac{1}{N}[{G}^{slab}(T,\,P,\,{N}_{Na},\,{N}_{Cl})-{N}_{Na}{\mu }_{Na}(T,\,P)-{N}_{Cl}{\mu }_{Cl}(T,\,P)],$$

where *γ*(*T*, *P*) is the surface energy per surface area, *G*^*slab*^(*T*, *P*, *N*_*Na*_, *N*_*Cl*_) is the Gibbs free energy of the surface per cell, *N*_*Na*_, *μ*_*Na*_ and *N*_*Cl*_, *μ*_*Cl*_ are the number and chemical potential of the Na and Cl atoms in the cell, respectively.

The chemical potentials in equilibrium with the NaCl substrate are related in the following way:

5 $${\mu }_{Na}(T,P)+{\mu }_{Cl}(T,P)={G}_{NaCl}(T,P),$$

where *G*_*NaC*_(*T*, *P*) is the Gibbs free energy of the bulk NaCl. Thus, surface energy can be expressed in a form with only one variable chemical potential:

6 $$\gamma (T,P)=\frac{1}{N}[{G}^{slab}(T,P,{N}_{Na},{N}_{Cl})-{N}_{Na}{G}_{NaCl}^{bulk}(T,P)-({N}_{Cl}-{N}_{Na}){\mu }_{Cl}(T,P)]$$

The lower limit of the chemical potential was set as the chemical potential of Cl at which Na deposits on the substrate, while the upper limit corresponds to the case where Cl_2_ molecules are saturated on the substrate. The following relation defines the physically meaningful range of chemical potentials:

7 $$\frac{1}{2}\Delta {E}_{f}(T,P)+\frac{1}{2}{E}_{C{l}_{2}}\le {\mu }_{Cl}(T,P)\le \frac{1}{2}{E}_{C{l}_{2}},$$

where ${E}_{C{l}_{2}}$ is the total energy of a chlorine molecule, $\Delta {E}_{f}(T,P)$ = 4.17 eV is the energy of formation of the bulk rock-salt NaCl, which is in close agreement with the experimental value of 3.98 eV at room temperature^67,68^.

Stability of different structures can be compared using Eq. (6) by plotting *γ* as a function of *μ*_*Cl*_, as shown in Figs 1b and 2b. Each structure corresponds to a line on the phase diagram. A complementary and equivalent way to determine stability is to plot the convex hull diagram (Figs 1c and 2c) in Δ*E*-Δ*N* axes^41^, where

8 $$\Delta E=\frac{1}{N}[{G}^{slab}(T,P,{N}_{Na},{N}_{Cl})-{N}_{Na}{G}_{NaCl}^{bulk}(T,P)]\,and\,\Delta N=\frac{1}{N}({N}_{Na}-{N}_{Cl})$$

## Supplementary information

Supplementary Information
