# Supplementary material for: Novel Unexpected Reconstructions of (100) and (111) Surfaces of NaCl: Theoretical Prediction
Source: Sci Rep. 2019 Oct 3;9:14267. doi: 10.1038/s41598-019-50548-8 (PMC6776646; doi:10.1038/s41598-019-50548-8)
Supplement: Supplementary file 1 — Supplementary Information [file 41598_2019_50548_MOESM1_ESM.docx]

Supporting Information for

Novel Unexpected Reconstructions of (100) and (111) Surfaces of NaCl. Theoretical Prediction

Alexander G. Kvashnin, ^1,2^ Dmitry G. Kvashnin, ^3,4^Artem R. Oganov, ^1,2,5^

^1^ Skolkovo Institute of Science and Technology, Skolkovo Innovation Center 143026, 3 Nobel Street, Moscow, Russia

^2^ Moscow Institute of Physics and Technology, 141700, 9 Institutsky Pereulok, Dolgoprudny, Russia

^3^ Emanuel Institute of Biochemical Physics RAS, 119334, 4 Kosigina st., Moscow, Russia

^4^ National University of Science and Technology MISiS, 119049, 4 Leninskiy prospect, Moscow, Russia

^5^ International Center for Materials Discovery, Northwestern Polytechnical University, Xi'an, 710072, China

Corresponding Authors

*Dr. D.G. Kvashnin, E-mail: [dgkvashnin@phystech.edu](mailto:dgkvashnin@phystech.edu)

Content

[Thermodynamic data of Cl2 molecule S2](#_Toc17457119)

[Wulff constructions S3](#_Toc17457120)

[References S4](#_Toc17457121)

## Thermodynamic data of Cl_2_ molecule

The Gibbs free energy *G* of the Cl_2_ molecule was calculated in the quasiharmonic approximation ^1^ as:

| $G\left( P,T \right)=E_{0}\left( V \right)+F_{vib}\left( T,V \right)+P\left( T,V \right)V$*,* | (1) |
| --- | --- |

where *E*_0_ is the total energy from the DFT calculations and *F_vib_* the vibrational Helmholtz free energy calculated as:

| $F_{vib}\left( T,V \right)=k_{B}T\int_{\Omega} g\left( \omega\left( V \right) \right)\ln\left[ 1-\exp\left( -\frac{\hbar\omega\left( V \right)}{k_{B}T} \right) \right]d\omega+\frac{1}{2}\int g\left( \omega\left( V \right) \right)\hbar\omega d\omega,$ | (2) |
| --- | --- |

where $g\left( \omega\left( V \right) \right)$ is the phonon density of states of a Cl_2_ molecule at the given volume, calculated using the density functional perturbation theory ^2^ as implemented in VASP. ^3–5^

We also take into account the rotational entropy *S_rot_* of Cl_2_ molecule, which will be included to the Gibbs free energy and can be calculated in the following way ^6^:

| $S_{rot}=R\cdot\left( \ln I+\ln T-\ln\sigma\right),$ | (3) |
| --- | --- |

where *I* is the moment of inertia, *σ* is the symmetry number.

Table S1. The comparison of experimental and calculated data on thermodynamic potentials of Cl_2_ molecule. H is standard enthalpy, S is standard entropy, G is Gibbs free energy. Reference values were taken from Ref. ^7^.

| *T*, K | *H_exp_*, kJ/mol | *S_exp_*, J/mol·K | *G_exp_*, kJ/mol | *H_calc_*, kJ/mol | *S_calc_*, J/mol·K | *G_calc_*, kJ/mol |
| --- | --- | --- | --- | --- | --- | --- |
| 300 | 0.06264 | 223.29 | -66.92 | 0.93110 | 224.81 | -66.51 |
| 400 | 3.53320 | 233.26 | -89.77 | 4.36314 | 236.39 | -90.19 |
| 500 | 7.10397 | 241.23 | -113.51 | 7.58677 | 243.15 | -113.99 |
| 600 | 10.73607 | 247.85 | -137.97 | 11.12205 | 249.90 | -138.82 |
| 700 | 14.40846 | 253.51 | -163.05 | 14.69785 | 255.69 | -164.29 |
| 800 | 18.10840 | 258.45 | -188.65 | 18.20515 | 260.51 | -190.21 |
| 900 | 21.82835 | 262.83 | -214.72 | 21.63526 | 264.37 | -216.30 |
| 1000 | 25.56463 | 266.77 | -241.20 | 25.27475 | 268.23 | -242.96 |

## Wulff constructions


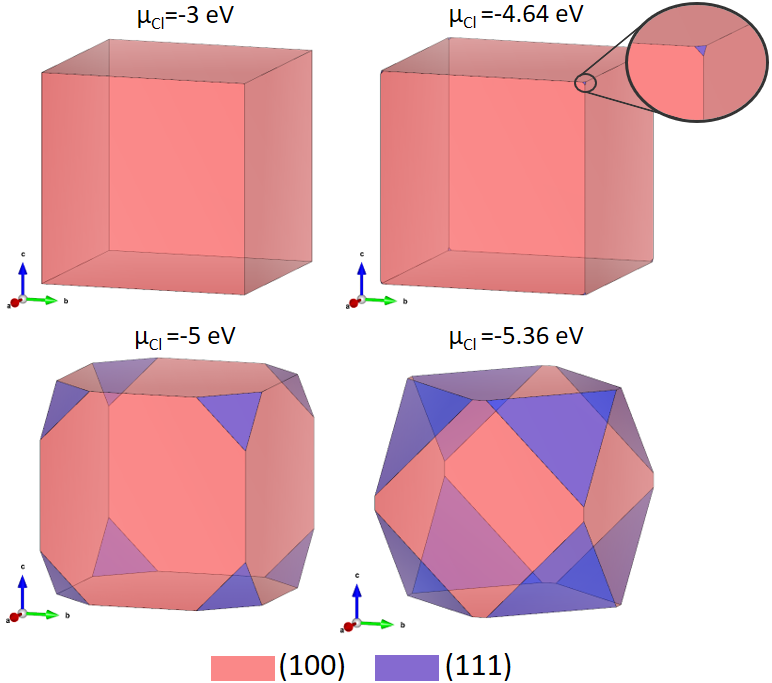


**Figure S1.** Wulff constructions of NaCl crystals at different values of chlorine chemical potentials.

## References

(1) Kern, G.; Kresse, G.; Hafner, J. Ab Initio Calculation of the Lattice Dynamics and Phase Diagram of Boron Nitride. *Phys Rev B* **1999**, *59*, 8551–8559. https://doi.org/10.1103/PhysRevB.59.8551.

(2) Baroni, S.; Giannozzi, P.; Testa, A. Green’s-Function Approach to Linear Response in Solids. *Phys. Rev. Lett.* **1987**, *58* (18), 1861–1864. https://doi.org/10.1103/PhysRevLett.58.1861.

(3) Kresse, G.; Hafner, J. Ab Initio Molecular Dynamics for Liquid Metals. *Phys. Rev. B* **1993**, *47* (1), 558–561.

(4) Kresse, G.; Hafner, J. Ab Initio Molecular-Dynamics Simulation of the Liquid-Metal-Amorphous-Semiconductor Transition in Germanium. *Phys. Rev. B* **1994**, *49* (20), 14251–14269.

(5) Kresse, G.; Furthmüller, J. Efficient Iterative Schemes for Ab Initio Total-Energy Calculations Using a Plane-Wave Basis Set. *Phys. Rev. B* **1996**, *54* (16), 11169–11186.

(6) Wei, J. Molecular Symmetry, Rotational Entropy, and Elevated Melting Points. *Ind. Eng. Chem. Res.* **1999**, *38* (12), 5019–5027. https://doi.org/10.1021/ie990588m.

(7) NIST, Chemistry WebBook http://webbook.nist.gov/.
